# Prevalence and antimicrobial resistance of *Salmonella *isolated from lactating cows and in contact humans in dairy farms of Addis Ababa: a cross sectional study

**DOI:** 10.1186/1471-2334-11-222

**Published:** 2011-08-19

**Authors:** Zelalem Addis, Nigatu Kebede, Zufan Worku, Haile Gezahegn, Alehegne Yirsaw, Tesfu Kassa

**Affiliations:** 1 University of Gondar, College of Medicine and Health Science, Department of Medical Laboratory Science, P.O.Box 196, Gondar, Ethiopia; 2 Addis Ababa University, Aklilu Lemma Institute of Pathobiology, P.O.Box 1176, Addis Ababa, Ethiopia; 3 National Animal Health Diagnostic and Investigation Center Research Center (NAHDIC), P.O.Box 04, Sebeta, Ethiopia

## Abstract

**Background:**

*Salmonella *are the major pathogenic bacteria in humans as well as in animals. *Salmonella *species are leading causes of acute gastroenteritis in several countries and salmonellosis remains an important public health problem worldwide, particularly in the developing countries. The situation is more aggravated by the ever increasing rate of antimicrobial resistance strains. Cattle have been implicated as a source of human infection with antimicrobial resistant *Salmonella *through direct contact with livestock and through the isolation of antimicrobial resistant *Salmonella *from raw milk, cheddar cheese, and hamburger meat traced to dairy farms. Despiite the presence of many studies on the prevalence and antimicrobial susceptibility pattern of *Salmonella *in Ethiopia, nothing has been said on the degree of the situation among apparently healthy lactating cows and in contact humans. Hence this study was conducted to determine the prevalence and antimicrobial resistance pattern of *Salmonella *isolates from lactating cows and in contact humans in dairy farms of Addis Ababa.

**Methods:**

a cross sectional study was conducted in Addis Ababa by collecting milk and faecal samples from lactating cows and stool samples from humans working in dairy farms. Samples were pre-enriched in buffered peptone water followed by selective enrichment using selenite cysteine and Rapaport-Vassilidis broths. Isolation and identification was made by inoculating the selectively enriched sample on to Xylose Lysine Deoxycholate agar followed by confirmation of presumptive colonies using different biochemical tests. The Kibry Bauer disk diffusion method was used for antimicrobial sensitivity testing.

**Results:**

10.7% (21/195) of cows and 13.6% (3/22) of the human subjects sheded *Salmonella*. 83% resistance to two or more antimicrobials and 100% resistance to ampicillin were observed. Most of the isolates were relatively sensitive to ciprofloxacin, cotrimoxazole, and chloramphenicol.

**Conclusion:**

High proportion of Salmonella isolates developed resistance to the commonly prescribed antimicrobials and this may be a considerable risk in the treatment of clinical cases. So, wise use of antimicrobials must be practiced to combat the ever increasing situation of antimicrobial resistance.

## Background

*Salmonella *are the major pathogenic bacteria in humans as well as in animals. *Salmonella *species are leading causes of acute gastroenteritis in several countries and salmonellosis remains an important public health problem worldwide, particularly in the developing countries [1]. Salmonellosis is the most common food borne disease in both developing and developed countries, although incidence rates vary according to the country [2]. The fecal wastes from infected animals and humans are important sources of bacterial contamination of the environment and the food chain [3].

Antimicrobial-resistant *Salmonella *are increasing due to the use of antimicrobial agents in food animals at sub-therapeutic level or prophylactic doses which may promote on-farm selection of antimicrobial resistant strains and markedly increase the human health risks associated with consumption of contaminated meat products [4-6]. Cattle have been implicated as a source of human infection with antimicrobial resistant *Salmonella *through direct contact with livestock and through the isolation of antimicrobial resistant *Salmonella *from raw milk, cheddar cheese, and hamburger meat traced to dairy farms. Antimicrobial use in animal production systems has long been suspected to be a cause of the emergence and dissemination of antimicrobial resistant *Salmonella *[7].

Different studies conducted in Ethiopia indicated considerable prevalence of *Salmonella *both in veterinary and public set ups [4,5,8,9] but reports from apparently healthy lactating cows and humans attending these cows is very limited. So the aim of this study was to determine the prevalence and antimicrobial susceptibility pattern of *Salmonella *isolates from apparently healthy lactating cows and in contact humans in dairy farms of Addis Ababa.

## Methods

### Sample Size Determination and Sample Collection Technique

The study was conducted from February, 2010 up to May, 2010 in Addis Ababa which has a bovine population of 58,568 [10].

Sample size was determined using prevalence rate of 7.1% from previous studies [8] at 5% level of significance and the following formula was employed [11]

$$\text{N}=\frac{{\left({\text{Z}}_{\alpha ∕2}\right)}^{2}\times \text{P}\left(1-\text{P}\right)}{{\text{d}}^{2}}$$

Based on the above formula the calculated sample size was 201.

A total of 195 lactating cows and 22 humans from 23 farms were randomly selected and included in this study. A total of 390 samples from cows (195 faecal and 195 milk) and 22 stool samples from humans were collected for the detection of *Salmonella*. The faecal specimens of cows were collected in a clean sterile air tight stool cup directly from the rectum. Approximately 20 ml of milk was collected in a sterile universal bottle after the cows were restrained in self-locking stanchions. 30 gms of stool samples, those do not have direct contact with the environment were collected in sterile stool cup with an applicator stick from volunteer individuals working in the dairy farms. The samples were transported using an ice box and analyzed at Aklilu Lemma Institute of Pathobiology, Addis Ababa University.

### Isolation and Identification of *Salmonella*

The isolation and identification of *Salmonella *was performed at the medical microbiology laboratory of Aklilu Lemma Institute of Pathobiology using techniques recommended by International Organizations for Standardization (ISO-6579, 2000), and those recommended by the Global *Salmonella *Surveillance (GSS) and National Health Services for Wales (NHS) [12-14]. The isolation and identification involves three steps; 1 gm of faecal sample or 1 ml of milk was pre-enriched with 9 ml of buffered peptone water (BPW) (Oxoid CM509, Basingstoke, England) and incubated for 24 hrs at 37°C. A portion (0.1 ml) of the pre-enriched cultured was transferred to 10 ml of selenite cysteine (SC) (Himedia M025, Mumbi) broth and another 0.1 ml portion was transferred to10 ml of Rappaport and Vassilidis (RV) broth (Merk, Darmstadt, Germany) broth and incubated at 37°C and 42°C for 24 hrs respectively. Finally, from the selective enrichment media the sample was inoculated on to Xylose Lysine Deoxycholate (XLD) (Oxoid CM0469, Basingstoke, England) agar and incubated at 37°C for 24 hrs and the incubation was prolonged to 48 hrs for those that did not show any growth during the 24 hrs incubation. Characteristic *Salmonella *colonies, having a slightly transparent zone of reddish color and a black center, were sub-cultured on nutrient agar (Oxoid CM0003, Basingstoke, England) and confirmed biochemically using triple sugar iron agar (TSI) (Oxoid CM0277, Basingstoke, England), Christensen's urea agar (Oxoid CM53, Basingstoke, England), lysine iron agar (LIA) (Oxoid CM381, Basingstoke, England), Voges Proskauer (VP), methyl red (MR) (Micromaster Thane, India), and Indole tests (Becton Dickinson, USA) [12].

### Antimicrobial susceptibility test of isolates

The antimicrobial susceptibility test of the isolates were performed according to the National Committee for Clinical Laboratory Standards (NCCLS) method using Kibry-Bauer disk diffusion test on Mulle-Hinton agar (Oxoid CM0337 Basingstoke, England) [15]. *Eshercia coli ATCC 25922 *was used as a quality control organism for the antimicrobial susceptibility test (Hendriksen, 2002). The isolates were tested for the following antibiotics; ampicillin (10 μg), nitrofurantoine (300 μg), streptomycin (10 μg), kanamycin (30 μg), gentamycin (10 μg), ceftriaxone (30 μg), chloramphenicol (30 μg), tetracycline (30 μg), ciprofloxacin (5 μg) all from Oxoid, England and cotrimoxazole (Thrimethoprim sulfmethoxazole) (25 μg) (Micromaster, India).

### Data Analysis

Data was analyzed using SPSS version 13 computer software (SPSS 13.0 Command Syntax Reference. SPSS Inc., Chicago, 2004) and presented in tables and graphs. The Chi-square test was utilized to assess significant differences in antimicrobial resistance of *Salmonella *isolates from human and cow and from isolates of milk and faeces of cows. A difference was taken as significant at a p-value less than 0.05.

**Ethical consideration: **the study was ethically approved by the Institutional Review Board of Aklilu Lemma Institute of Pathobiology, Addis Ababa University. More over both informed and written consent were obtained from the human subjects.

## Result

From 23 farms included in this study detection of *Salmonella *was successful in 11 (47.8%) dairy farms. The relative prevalence within different farms was 57.1% (4/6), 33.33% (2/6) and 83.33% (5/6) for small, medium and large scale farms, respectively.

From the total of 195 dairy cows tested, 10.76% (21/195) were positive for *Salmonella*, either from milk or faeces. Of these cows, 71.4% (15/21) were positive from faecal sample and 28.6% (6/21) were positive from milk sample. None of the cows were positive both from the faeces and milk sample, and no significant difference was found in the isolation of *Salmonella *from faecal sample and milk samples (x^2 ^= 0.516, P-value = 0.473). The stool samples were collected from the 22 volunteer individuals working in the dairy farms and the result showed that only 13.63% (3/22) of them became positive for *Salmonella *(Table 1).

**Table 1:** *Salmonella *Isolates from lactating dairy cows and individuals working in the dairy farms

Sample type	Address of farms		Farm size			*X^2^*(P- value)
	Bole	Yeka	Small	Medium	Large	
Faeces of cows						
Positive	5 (5.9%)	10 (9.1%)	2 (3.9%)	5(11.6%)	8(7.9%)	1.967 (0.374)
Negative	80(94.1%)	100 (90.9%)	49(96.1%)	38(88.4%)	93(92.1%)	
Total	85 (100%)	110 (100%)	51(100%)	43(100%)	101(100%)	
Milk of cows						
Positive	5(5.9%)	1(1%)	5(9.8%)	1(2.3%)	0(0%)	11.02 (0.004)
Negative	80(94.1%)	109(99%)	46(90.2%)	42(97.7%)	101(100%)	
Total	85(100%)	110(100%)	51(100%)	43(100%)	101(100%)	
Human stool						
Positive	2(20%)	1(8.3%)	0(0%)	1(25%)	2(15.4%)	1.262 (0.532)
Negative	8(80%)	11(91.7%)	5(100%)	3(25%)	11(84.6%)	
Total	10(100%)	12(100%)	5(100%)	4(100%)	13(100%)	

*Numbers in parenthesis are percentages*.

**Figure 1:**
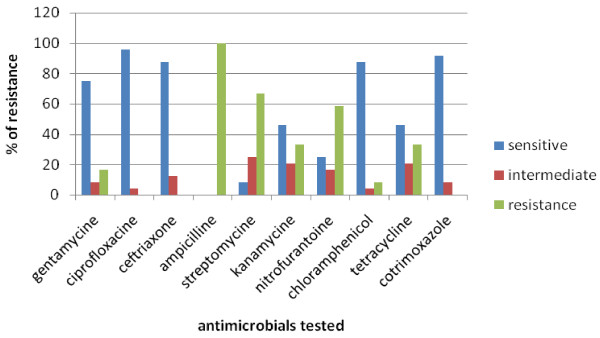
**Percentage activity of antimicrobials tested against *Salmonella *isolates from lactating dairy cows and individuals working in the dairy farms**.

All the twenty four isolates of *Salmonella*, from cows and humans, were subjected to a panel of ten antimicrobials. The antimicrobial susceptibility pattern of the isolates indicated that all isolates were 100%, 66.7%, and 58.3% resistant to ampicillin, streptomycin and nitrofurantoine respectively. On the other hand the isolates were, 91.7%, 87.5% and 75% sensitive to ciproflocacillin and cotrimoxazole, chloramphenicol and ceftriaxone respectively (Figure 1).

83.3% of both human and cow isolates showed resistance for two or more of the antimicrobials tesed. Form these resistance isolates, most of them (20%) showed resistance to ampicillin and streptomycin followed by resistance to gentamycin, ampicillin, streptomycin, kanamycin and nitrofurantoine (15%) and to ampicillin, streptomycin and nitrofurantoine (10%). One milk isolate and one faecal isolate of cow showed multiple antimicrobial resistances to 60% of the antimicrobials tested (Table 2).

**Table 2:** Multiple antimicrobial resistance profile of *Salmonella *isolates from lactating dairy cows and humans working in dairy farms

Number of antimicrobial resistance	Antimicrobial resistance pattern (number of isolates)	Number of isolates (%)
Two	AMP, S (4) AMP, TE (2)	6(25)
Three	AMP, S, F (2) AMP, S, TE (1) AMP, K, F (1)	4(16.7)
Four	AMP, S, K, F (1) CN, AMP, S, F (1) CN, AMP, K, F (1) AMP, S, F, TE (1)	4(16.7)
Five	CN, AMP, S, K, F (3) AMP, S, F, TE, C (1)	4 (16.7)
Six	CN, AMP, S, K, F, TE (1) AMP, S, K, F, TE, C (1)	2(8.3)

***Key to Abbreviations***: AMP (ampicillin), S (streptomycin), K (kamanycine), F (nitrofurantoine), CN (gentamycin), TE (tetracycline), C (chloramphenicol).

*Salmonella *isolated from milk sample showed 100% resistance to ampicillin and streptomycin while they were 83.3% sensitive to ciprofloxacin, cotrimoxazole and ceftriaxone. Faecal isolates of cows showed 100% sensitivity to ciprofloxacin followed by cotrimoxazole and chloramphenicol (93.3%). 66.66% resistance to streptomycin was observed among faecal isolates of cows. All the human isolates showed resistance only for ampicillin and they were sensitive to most antimicrobials tested with 100% intermediate resistance to streptomycin. Significance difference in the antimicrobial resistance of isolates from different samples was not observed (Table 3).

**Table 3:** Antimicrobial susceptibility pattern of *Salmonella *isolates from lactating dairy cows and humans working in dairy farms

Antimicrobials tested	Type of sample			*X^2 ^(P-value)*
	Milk of cows	Faeces of cows	Human stool	
Gentamycin				
Sensitive	4(66.7)	11(73.3)	3(100)	2.535(0.792)
Intermediate	0(0)	2 (13.3)	0(0)	
Resistance	2(33.3)	2 (13.3)	0(0)	
Ciprofloxacin				
Sensitive	5(83.3)	15(100)	3(100)	3.118(0.708)
Intermediate	1(16.7)	0(0)	0(0)	
Resistance	0(0)	0(0)	0(0)	
Ceftriaxone				
Sensitive	5(83.3)	13(86.7)	3(100)	0.664(1)
Intermediate	1(16.7)	2(13.3)	0(0)	
Resistance	0(0)	0(0)	0(0)	
Ampicilline				
Sensitive	0(0)	0(0)	0(0)	
Intermediate	0(0)	0(0)	0(0)	
Resistance	6(100)	15(100)	3(100)	
Streptomycin				
Sensitive	0(0)	2(13.3)	0(0)	9.422(0.083)
Intermediate	0(0)	3(20)	3(100)	
Resistance	6(100)	10(66.7)	0(0)	
Kanamycine				
Sensitive	2(33.3)	7(46.7)	2(66.7)	4.834(0.167)
Intermediate	0(0)	4(26.7)	1(33.3)	
Resistance	4(66.7)	4(26.7)	0(0)	
Nitrofurantoine				
Sensitive	2(33.3)	3(20)	2(66.7)	8.303(0.083)
Intermediate	0(0)	3(20)	1(33.3)	
Resistance	4(66.7)	9(40)	0(0)	
Tetracycline				
Sensitive	2(33.3)	8(53.3)	1(33.3)	2.061(0.875)
Intermediate	1(16.7)	3(20)	1(33.3)	
Resistance	3(50)	4(26.7)	1(33.3)	
Chloramphenicol				
Sensitive	4(66.7)	14(93.3)	3(100)	4.605(0.333)
Intermediate	1(16.7)	0(0)	0(0)	
Resistance	1(16.7)	1(6.7)	0(0)	
Cotrimoxazole				
Sensitive	5(83.3)	14(93.3)	3(100)	1.289 (0.792)
Intermediate	1(16.7)	1(6.7)	0(0)	
Resistance	0(0)	0(0)	0(0)	

*Numbers in parenthesis are percentages*.

## Discussion

In this study the prevalence of *Salmonella *in apparently healthy lactating dairy cows is larger (10.76%) as compared to other studies, even though most of the reports are on slaughtered cattle from abattoirs and ready to eat food items [6,8,9]. Hence lactating cows could be potential sources of *Salmonella *infection for individuals working in dairy farms and for the community at large.

Alemayehu et al., 2003 (8) reported a prevalence of 7.1% from apparently healthy slaughtered cattle which is less than the present report. This difference may be attributed to the difference in the tests used, since pre-enrichment steps using buffered peptone water was employed in this study. On the other hand reports from England (0.2% and 4%) and from Northern Thailand (3%) are much lower than the current investigation [16-18]. But a report from Cameroon by Akoachere et al., 2009 [19] indicated a very high prevalence (27%) of *Salmonella *among cattle. This may be due to the difference in the living condition, like housing conditions, feeding habits, types of feed given for the cattle, of the two cattle populations. A comparable result, 9.96%, was reported from four states of USA [20].

The prevalence of *Salmonella *among individuals working in dairy farms of Addis Ababa was 13.63%. The result is higher than a study conducted by Alemayehu et al., 2003 [8] and Zewdu and Cornelius, 2009 [6] who reported a prevalence of 6% and 7.6%, respectively. The difference may be due to different working environment, hence different hygienic status, of study subjects. This higher prevalence is a concern to the dairy farms that provide milk and milk products to the community since cross contamination from infected individuals could be a potential source of food borne infections.

Resistance for two or more of antimicrobials (83.3%) which was observed in this study was higher than other studies conducted in Ethiopia [5,6,8,21] and elsewhere in the world [1,2,22,23]. This difference may be due to the increasing rate of inappropriate utilization of antibiotics in the dairy farms which favors selection pressure that increased the advantage of maintaining resistance genes in bacteria [24,25].

Zewdu and Cornelius (2009) [6] reported that the isolates of *Salmonella *from food items and personnel from Addis Ababa were resistant to the commonly used antibiotics including streptomycin, ampicillin, and tetracycline. The result of the current research also indicated resistance of *Salmonella *isolates to commonly used antimicrobials including ampicillin, streptomycin, nitrofurantoine, kanamycine and tetracycline, with resistance rate of 100%, 66.7%, 58.3% and 33.3%, respectively.

All the isolated *Salmonella*, in the current study, were 100% resistant to ampiciliin. This finding is in line with previous reports from South India [26], from Nigeria [27] and from Cameroon [19] which reported a similar 100%, over 90% and 100% resistance to ampicillin, respectively. Hghi et al. (2009) [28] reported a resistance rate of 60.3% and72.7% in different study periods among human isolates from Iran, which is slightly lower than the current finding.

Ciprofloxacin showed a good antimicrobial activity against both human and cow isolates. This is also comparable with the result reported by Akinyemia et al., 2005 [27] from Nigeria, among human isolates and with that reported by Molla et al., 2006 [5] from central part of Ethiopia among isolates of sheep and goat. Though no data has indicated this, the effectiveness of such drugs like ciprofloxacin may be because they are not widely used in countries like Ethiopia and other African countries.

In the current study cotrimoxazole (trimethoprim-sulfametoxazole) showed a good antimicrobial activity against all isolates and no resistant isolate against this drug was detected. This result is lower than the reports by Rotimi et al., 2008 [1] from Kuwait and United Arab Emirates who reported a resistance rate of 26.1% and 8.9%, respectively. Even though cotrimoxazole has been widely available the reason of its effectiveness until this times need investigations.

## Conclusion

Investigating the prevalence and antimicrobial resistance of *Salmonella *from cattle and incontact human in dairy farms is of paramount importance to design methods of minimizing the possible transmission of *Salmonella *between humans and cattle. Moreover it important in combating the emergence of antibiotic resistant strains of *Salmonella*. The information gathered in this cross sectional study, together with other similar studies, is important to achieve the aforementioned importance of studying *Salmonella *in dairy farms.

In general from this cross sectional study it can be concluded that the prevalence of *Salmonella *in lactating cows and individuals working in dairy farms in Addis Ababa is 10.76% and 13.63% respectively. This result is significantly high to be a potential source of food borne salmonellosis. High proportion (83.3%) of *Salmonella *isolates were resistant to two or more of the antimicrobials that are commonly used in the veterinary and public health set up. This may pose difficulties in the treatment of human clinical cases and other bacterial diseases.

The currents study indicated the necessity of a further investigation on the prevalence and antimicrobial susceptibility pattern of *Salmonella*, by considering it as a potential food borne pathogen, starting from the farm to table. Molecular characterization of the isolates with emphasis on resistant strains is also necessary to identify mechanisms of antibiotic resistance. More over judicious and prudent use of antimicrobials in the veterinary and public health sectors is mandatory since high rate of antimicrobial resistant *Salmonella *isolates were identified.

## Competing interests

The authors declare that they have no competing interests.

## Authors' contributions

ZAM conceived the study, undertook statistical analysis and drafted the manuscript. NKW, ZSW, HAG, AWY and TK initiated the study and made major contributions to the study design and statistical analysis. All authors contributed to the writing of the manuscript and approved the submitted version of the manuscript.

## Pre-publication history

The pre-publication history for this paper can be accessed here:

http://www.biomedcentral.com/1471-2334/11/222/prepub
